# The Association between Serum 25-Hydroxy Vitamin D Level and Urine Cathelicidin in Children with a Urinary Tract Infection

**DOI:** 10.4274/jcrpe.2563

**Published:** 2016-09-01

**Authors:** Duygu Övünç Hacıhamdioğlu, Demet Altun, Bülent Hacıhamdioğlu, Ferhat Çekmez, Gökhan Aydemir, Mustafa Kul, Tuba Müftüoğlu, Selami Süleymanoğlu, Ferhan Karademir

**Affiliations:** 1 Gülhane Military Medical Academy, Haydarpaşa Training and Research Hospital, Clinic of Pediatrics, İstanbul, Turkey; 2 Etimesgut Military Hospital, Clinic of Pediatrics, Ankara, Turkey; 3 Gülhane Military Medical Academy, Haydarpaşa Training and Research Hospital, Clinic of Biochemistry, İstanbul, Turkey

**Keywords:** Urinary tract infection, Escherichia coli, children, Vitamin D, cathelicidin

## Abstract

**Objective:**

Cathelicidin is an important antimicrobial peptide in the urinary tract. Cathelicidin expression is strongly stimulated by 1,25-dihydroxy vitamin D in epithelial cells, macrophages/monocytes, and neutrophils. Vitamin D and cathelicidin status in children with urinary tract infection (UTI) caused by Escherichia coli is unknown. To establish the relationship between serum vitamin D and urine cathelicidin levels in children with a UTI caused by Escherichia coli.

**Methods:**

Serum 25-hydroxy vitamin D and urine cathelicidin levels were measured in 36 patients with UTI (mean age 6.8±3.6 years, range: 0.25-12.6 years) and 38 controls (mean age 6.3±2.8 years, range: 0.42-13 years).

**Results:**

There were no significant differences in urine cathelicidin levels between the study and control groups (p>0.05). Eight (22.2%) patients in the study group and 21 (58.3%) children in the control group were found to have sufficient vitamin D (≥20 ng/mL). Patients with sufficient vitamin D had higher urine cathelicidin levels than the controls with sufficient vitamin D (respectively 262.5±41.1 vs. 168±31.6 ng/mL, p=0.001). There were no significant differences between the patients and controls with insufficient vitamin D (p>0.05).

**Conclusion:**

The children with vitamin D insufficiency may not be able to increase their urine cathelicidin level during UTI caused by Escherichia coli. There is a need of prospective studies in order to prove a beneficial effect of vitamin D supplementation for the restoration of cathelicidin stimulation and consequently for prevention of UTI recurrence.

**WHAT IS ALREADY KNOWN ON THIS TOPIC?**

A vitamin D insufficiency can affect the defense system through multiple pathways and lead to infection. Cathelicidin plays many roles protecting the urinary tract from infection. Cathelicidin expression is strongly stimulated by 1,25-dihydroxy vitamin D in epithelial cells, macrophages/monocytes, and neutrophils.

**WHAT THIS STUDY ADDS?**

The children with vitamin D insufficiency may not be able to increase their urine cathelicidin level during urinary tract infection caused by Escherichia coli.

## INTRODUCTION

Urinary tract infection (UTI) is one of the most commonly acquired bacterial infections in childhood. It affects 10% of children and causes significant morbidity (1). Escherichia coli (E. coli) is the predominant pathogen in childhood UTI found in 90% of girls and in 80% of boys at the primary UTI. An important factor for the predominance of E. coli is their ability to attach to the urinary tract endothelium (2).

Vitamin D deficiency and insufficiency is a global issue (3). It is well known that vitamin D is involved in classical calcium homeostasis. More recent data from a variety of sources indicate that vitamin D has a broad spectrum of actions against autoimmune diseases and infections (4,5). It was shown that recurrent UTIs in premenopausal women are associated with vitamin D deficiency (6). Recently, it was reported that serum 25-hydroxy vitamin D level of <20 ng/mL was associated with UTI in children (7). Furthermore, the vitamin D receptor gene polymorphism was an important factor for UTI susceptibility in a study of children diagnosed with UTI and in this study, the most commonly isolated agent from urinary cultures was E. coli (8). Although it is not yet known the mechanism between the vitamin D deficiency and infection, a vitamin D insufficiency can affect the defense system through multiple pathways and lead to infection. Unfortunately, we did not evaluate all of the factors in the urinary tract defense system with which vitamin D is associated.

It has been speculated that urinary tract defense may be profoundly dependent on specific soluble epithelial cell-derived mediators and one of them are inducible bactericidal antimicrobial peptides, such as α- and β-defensins and cathelicidin (9,10). Among them, cathelicidin is involved in protecting the urinary tract from infection. Cathelicidin may stimulate production of chemokines and cytokines by several cell types and it also plays an important role in maintaining urinary tract integrity (11,12,13). Furthermore, cathelicidin expression is strongly stimulated by 1,25-dihydroxy vitamin D in epithelial cells, macrophages/monocytes, and neutrophils (11).

Vitamin D and cathelicidin status in children with UTI is unknown. Therefore, in this study, we analyzed serum vitamin D and urine cathelicidin levels in children with a UTI caused by to E. coli.

## METHODS

### Patients

This study was approved by the local ethics committee and performed in accordance with the Declaration of Helsinki. Informed consent was obtained. Participants were recruited in the Pediatric Department from April to July 2014. The study group consisted of children with a UTI. The control group consisted of 38 healthy, age- and sex-matched children. Exclusion criteria were (i) prior UTI history; (ii) congenital anatomical anomalies such as meningomyelocele, cardiac abnormalities requiring surgery, skeletal dysplasia, renal hypo-dysplasia, cystic kidney disease, midline defects; (iii) chronic diseases and drug use such as to treat diabetes mellitus, epilepsy, bronchial asthma, chronic renal failure, hypertension; (iv) previous infections in the 6 weeks before the study; (v) obesity or malnutrition (vi) permanent urinary catheter, urinary tract stent or nephrostomy tube, urinary incontinence, neurogenic bladder; (vii) asymptomatic bacteriuria; (viii) kidney malformations and kidney stones; (ix) vesicoureteral reflux (VUR); and (x) chronic drug use or vitamin D supplementation. Diagnostic criteria for an upper UTI were fever, at least one UTI symptom (flank pain or costovertebral tenderness), pyuria, and presence of E. coli in the urine. Diagnostic criteria for a lower UTI were at least one UTI symptom (dysuria, urgency, suprapubic pain, irritability), pyuria, and presence of E. coli in the urine. We defined a pyuria as ≥5 white blood cells (WBCs) per high-power field on a spun urine. Urine samples were obtained by the midstream clean catch method for toilet-trained children, by urinary catheters for febrile infants and small children, and by bag for afebrile infants and small children. Presence of ≥50.000 cfu/mL of E. coli by catheterization or ≥105 cfu/mL E. coli by mid flow urine were considered indicative of a positive urine culture (14). In a bag sample, if the urinalysis result was positive for pyuria in a symptomatic patient and there was a single organism cultured with a colony count >100,000, UTI was diagnosed (15).

All patients underwent renal and bladder ultrasonography within 48 hours of admission. A voiding cystourethrogram (VCUG) was considered to be indicated if renal and bladder ultrasonography revealed hydronephrosis or other findings that would suggest either high-grade VUR or obstructive uropathy. Other atypical or complex clinical or laboratory findings (poor urine flow, abdominal or bladder mass, raised creatinine level, septicemia, failure to respond to correct antibiotic treatment within 48 h, presence of VUR in first-degree family members, infection with non-E. coli organisms) were also accepted as indications for VCUG (16,17).

### Study Design

This was a cross-sectional prospective study that examined the association between 25-hydroxy vitamin D and urine cathelicidin levels in children with UTI and in a healthy control group. Serum and urine samples were obtained before UTI treatment. Demographic variables (age and sex), disease type (lower or upper UTI), results of urinalysis, blood levels for C-reactive protein, white blood cell count, serum vitamin D, and urine cathelicidin levels were recorded. The serum samples were protected from light and stored at -80 °C prior to analysis. Serum 25-hydroxy vitamin D levels were measured using a commercial enzyme-linked immunosorbent assay (ELISA) kit (DIAsource, Louvain-la Neuve, Belgium).

Vitamin D deficiency has been defined by the Institute of Medicine (IOM) as a measured serum 25-hydroxy vitamin D <12 ng/mL, vitamin D insufficiency when serum 25-hydroxy vitamin D is between 12 and 20 ng/mL, and vitamin D sufficiency is defined as a 25-hydroxy vitamin D level of 20 ng/mL (18).

Clean urine samples were taken midstream and in bags. Urine samples were centrifuged for 20 min at 2000-3000 rpm, and the supernatant was removed. The specimens were stored at -80 °C prior to analysis. Urine cathelicidin levels were measured using a commercial ELISA kit (Eastbiopharm, Hangzhou, China). The kit uses a double-antibody sandwich ELISA to assay the human cathelicidin-1 level in the sample. Cathelicidin-1 was added to wells pre-coated with human cathelicidin-1 monoclonal antibody. After incubation, biotin-labeled cathelicidin-1 antibody was added and combined with streptavidin- horseradish peroxidase to form an immune complex. The color change, known to be positively correlated with the human cathelicidin-1 concentration in the sample, was measured spectrophotometrically at 450 nm. The intra-assay coefficient of variation (CV) was <10%, and the inter-assay CV was <12%. The assay range was 7-300 ng/mL, and sensitivity was 3.21 ng/mL.

### Statistical Analysis

All statistical calculations were carried out using SPSS for Windows ver. 15.0 (SPSS, Inc., Chicago, IL, USA). Simple between-group comparisons were made using Student’s t-test. The parameters are expressed as mean values and standard deviations. Spearman’s rank test was used for the correlation analysis. A p-value <0.05 was considered statistically significant.

## RESULTS

The study group consisted of 36 children (6 males; mean age, 6.8±3.6 years; range, 0.25-12.6 years). Thirty-eight children (10 males; mean age, 6.3±2.8 years; range, 0.42-13 years) served as the control group. No significant differences in age or sex were observed between the study and control groups. Vitamin D levels differed significantly between the groups (p<0.05); however, no differences in urine cathelicidin levels were detected. The demographic and biochemical values of the study and control groups are presented in Table 1.

Eleven (30.5%) patients had an upper UTI (2 males; mean age, 4.7±2.5 years; range, 0.25-10 years) and 25 (69.4%) patients had a lower UTI (4 males; mean age, 7.4±3.2 years; range, 1.1-12 years). There were significant differences in age between patients with upper and those with lower UTIs (p=0.013). No significant differences were observed between patients with upper and lower UTI in gender, vitamin D and cathelicidin levels, or presence of pyuria. There was no correlation between the urine cathelicidin and pyuria level in the patient group (p=0.794, r=-0.045).

Eight (22.2%) patients in the study group and 21 (58.3%) children in the control group had sufficient vitamin D levels (≥20 ng/mL). Twelve (33.3%) patients in the study group and 15 (39.5%) children in the control group had vitamin D levels between 12-19 ng/mL. Sixteen (44.4%) patients in the study group and two (5%) children in the control group had vitamin D deficiency (<12 ng/mL). Patients with an adequate vitamin D level (n=8) had a higher mean urine cathelicidin level than did the controls with sufficient vitamin D (n=21) (p=0.001). There were no significant differences for urine cathelicidin level between the patient and control groups with insufficient vitamin D (p>0.05). The demographic and cathelicidin values of these patients are presented in Table 2. There was no correlation between the urine cathelicidin level and pyuria in the patient group with insufficient vitamin D nor in those with sufficient vitamin D (p>0.05).

Using chi-square test (vitamin D insufficient/sufficientxUTI/control), findings revealed there was a dependency relationship between vitamin D status and UTI (x2counted=7.139, x2expected=14.11, SD=1, p=0.008).

**Figure 1:**
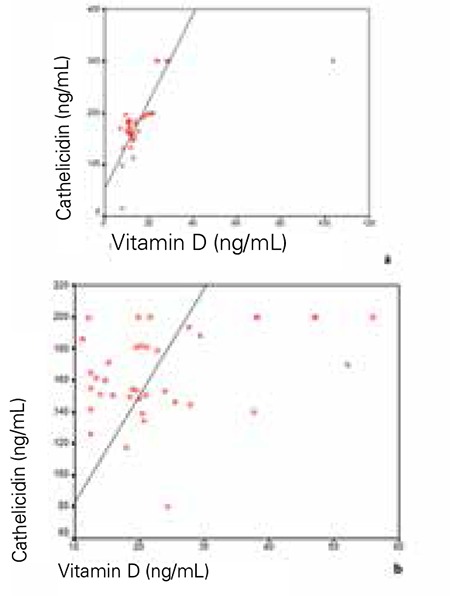
(a, b) Correlations between vitamin D and urine cathelicidin level. a) There was a positive correlation between vitamin D and urine cathelicidin levels in the study group (n=36, p<0.0001, r=0.587). b) There was a positive correlation between vitamin D and urine cathelicidin level in the control group (n=38, p=0.02, r=0.367)

A positive correlation was found between vitamin D and urine cathelicidin levels in the study (n=36, p<0.0001, r=0.587, Figure 1a) and control groups (n=38, p=0.02, r=0.367, Figure 1b).

Cathelicidin levels were higher in the vitamin D sufficient (n=29) group as compared to the vitamin D insufficient (n=45) group. These levels were respectively 169.17±28 vs. 153.87±32 ng/mL, p=0.038 in these two groups. A positive correlation was found between vitamin D and urine cathelicidin levels in the vitamin D sufficient group (p=0.022, r=0.307). However, there was no correlation between vitamin D and urine cathelicidin levels in the vitamin D insufficient group (p=0.372).

## DISCUSSION

Effects of vitamin D beyond the skeletal system are becoming increasingly important. In this study, we examined the relationship between serum levels of vitamin D and urinary cathelicidin for the first time in children with a UTI. We found that urine cathelicidin level did not increase significantly during a UTI in children with vitamin D insufficiency.

Recently, it was reported that a serum 25-hydroxy vitamin D level of <20 ng/mL was associated with UTI in children (7). In our study, frequency of vitamin D insufficiency was significantly higher in children with a UTI than in those in the control group. In a study conducted on premenopausal women, it was found that uncomplicated UTI caused by E. coli lead to an increase in urinary cathelicidin levels during infection compared to postinfection levels (19). It was also shown that cathelicidin expression and secretion were increased during E. coli urinary tract colonization in children with cystitis or pyelonephritis (12). However, we found no differences in the cathelicidin levels between the study and control groups which may have been associated with the state of vitamin D insufficiency in this group of subjects. When the groups were divided according to their vitamin D levels, patients with sufficient vitamin D levels had higher cathelicidin levels than the controls who also were vitamin D sufficient. On the other hand, there were no significant differences in urine cathelicidin level between patients with insufficient vitamin D and the controls with insufficient vitamin D. According to these findings, vitamin D insufficiency did not lead to an increase in the urine cathelicidin level during UTI in these children. These results suggest that sufficient vitamin D may be required to increase urine cathelicidin levels during a UTI.

Our findings revealed there is a dependency relationship between vitamin D status and UTI. Although this relationship is not exactly defined, the 25-hydroxy vitamin D is known to have an effect on the urothelium, with immunomodulatory capacity against E. coli infection (11,12,20). Recently, it was demonstrated that during pregnancy, increases in 25-hydroxy vitamin D and cathelicidin levels were observed as pregnancy advanced and, as gestation advanced, serum had an increased capacity to inhibit E. coli growth in urothelial cells (21). In a study of postmenopausal women, after vitamin D supplementation, increased cathelicidin expression in bladder biopsy samples with E. coli infection was observed compared with prior to supplementation (20). In this present study, we also established a positive correlation between vitamin D and cathelicidin in vitamin D-sufficient children. These results suggest that the effect of serum vitamin D on cathelicidin expression in the urinary tract depends on the level of vitamin D.

The major sources of cathelicidin in the urinary tract are circulating neutrophils, renal cells, and uroepithelial cells (12). A positive correlation has previously been observed between cathelicidin level and pyuria (12). However, we did not find a similar correlation in sufficient and insufficient vitamin D patients. In a study of cathelin-related antimicrobial peptide (CRAMP; an ortholog of the sole human cathelicidin) deficient mice, it was found that CRAMP-deficient hosts demonstrated less intense cytokine responses, diminished neutrophil infiltration, and accelerated uroepithelial recovery (22). Thus, it would seem that CRAMP may enhance E. coli infection in the bladder by promoting local inflammation. However, the authors pointed out that in total, their data indicate activities for cathelicidin during UTI that are independent of its direct antimicrobial activity. It appears that we need more studies for multiple biological activities during host-pathogen interactions.

It was recently reported that the frequency of vitamin D insufficiency in Turkish children and adolescents was 40% (23). The frequency of vitamin D insufficiency was similar in our control group.

Accumulated knowledge about cathelicidin and its relationship with urinary infection has been associated with E. coli. We therefore, in this study, preferred to include patients with UTI caused by E. coli to ensure homogeneity.

This study had some limitations. The sample size was low. This was a cross-sectional study; therefore, we do not know the previous urine cathelicidin status of the patients. A prospective study is needed to examine the beneficial effect of vitamin D supplementation to restore cathelicidin expression and prevent UTI recurrence.

The recommended vitamin D doses support bone health but also seem to be useful for fighting infection. Our results suggest that adequate vitamin D may benefit the urinary tract during a UTI by inducing cathelicidin expression.

In conclusion, our main finding was that the urine cathelicidin level was significantly upregulated in children with UTI and sufficient vitamin D status. In contrast, urine cathelicidin levels did not increase significantly during a UTI in children who had vitamin D insufficiency. Vitamin D deficiency and insufficiency is a global issue. Determining the vitamin D status of children with a UTI history and supplementing to restore proper vitamin D levels is a simple and cheap approach. The vitamin D-cathelicidin pathway is a human/primate-specific process; therefore, additional human studies should reveal the appropriate vitamin D level that is most beneficial during a UTI.

## Ethics

Ethics Committee Approval: This study was approved by the local ethics committee and performed in accordance with the Declaration of Helsinki, Informed Consent: Informed consent was obtained.

Peer-review: Externally peer-reviewed.

## Figures and Tables

**Table 1:**
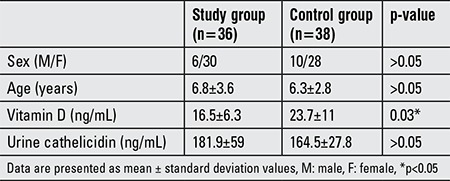
Demographic and biochemical characteristics of the study and control groups

**Table 2:**
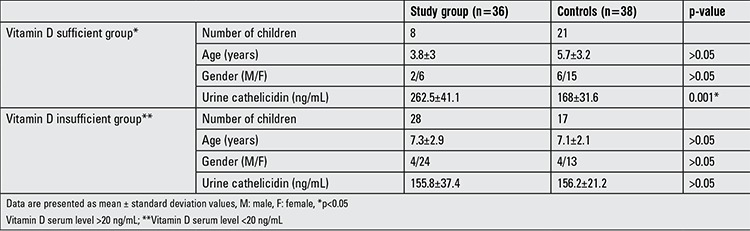
Demographic characteristics and cathelicidin values of the groups according to vitamin D status
